# Use of a novel microbiome modulator improves anticancer immunity in a murine model of malignant pleural mesothelioma

**DOI:** 10.1016/j.xjon.2024.02.007

**Published:** 2024-02-17

**Authors:** Christophe Gattlen, Kirby R. Frank, Damien N. Marie, Aurélien Trompette, Louis-Emmanuel Chriqui, Yameng Hao, Etienne Abdelnour, Michel Gonzalez, Thorsten Krueger, Paul J. Dyson, Sviatlana Siankevich, Christophe von Garnier, Niki D.J. Ubags, Sabrina Cavin, Jean Y. Perentes

**Affiliations:** a Division of Thoracic Surgery, Department of Surgery, CHUV, Lausanne University Hospital and University of Lausanne, Lausanne, Switzerland; b Division of Pulmonology, Department of Medicine, CHUV, Lausanne University Hospital and University of Lausanne, Lausanne, Switzerland; c Institute of Chemical Sciences and Engineering, Swiss Federal Institute of Technology (EPFL), Lausanne, Switzerland; d Embion Technologies SA, Etoy, Switzerland

**Keywords:** malignant pleural mesothelioma, prebiotic, microbiome, antitumor immunity, microbiome modulator

## Abstract

**Objective:**

Malignant pleural mesothelioma is a fatal disease and a clinical challenge, as few effective treatment modalities are available. Previous evidence links the gut microbiome to the host immunoreactivity to tumors. We thus evaluated the impact of a novel microbiome modulator compound (MMC) on the gut microbiota composition, tumor immune microenvironment, and cancer control in a model of malignant pleural mesothelioma.

**Methods:**

Age- and weight-matched immunocompetent (n = 23) or athymic BALB/c mice (n = 15) were randomly assigned to MMC or no treatment (control) groups. MMC (31 ppm) was administered through the drinking water 14 days before AB12 malignant mesothelioma cell inoculation into the pleural cavity. The impact of MMC on tumor growth, animal survival, tumor-infiltrating leucocytes, gut microbiome, and fecal metabolome was evaluated and compared with those of control animals.

**Results:**

The MMC delayed tumor growth and significantly prolonged the survival of immunocompetent animals (*P* = .0015) but not that of athymic mice. The improved tumor control in immunocompetent mice correlated with increased infiltration of CD3^+^CD8^+^GRZB^+^ cytotoxic T lymphocytes in tumors. Gut microbiota analyses indicated an enrichment in producers of short chain fatty acids in MMC-treated animals. Finally, we observed a positive correlation between the level of fecal short chain fatty acids and abundance of tumor-infiltrating cytotoxic T cells in malignant pleural mesothelioma.

**Conclusions:**

MMC administration boosts antitumor immunity, which correlates with a change in gut microbiome and metabolome. MMC may represent a valuable treatment option to combine with immunotherapy in patients with cancer.

## Graphical Abstract

**Figure:**
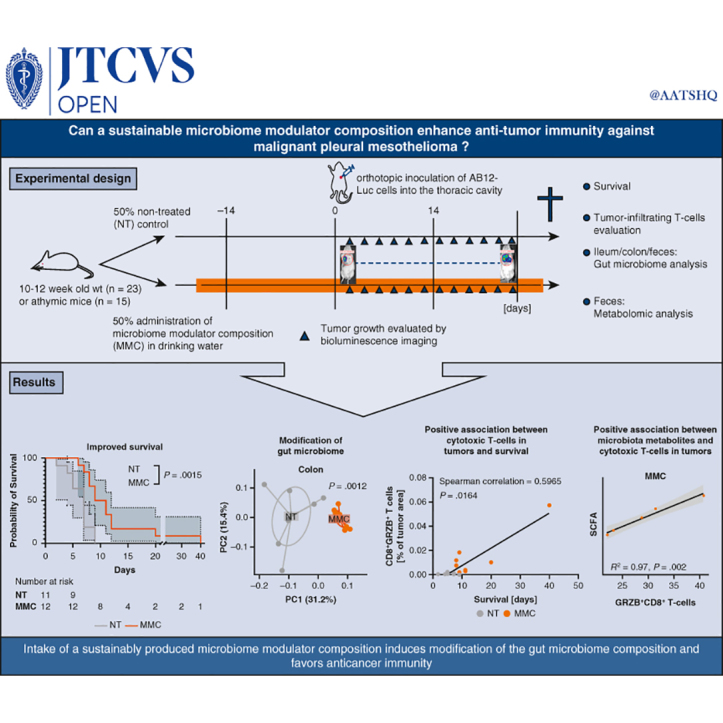

**Figure:**
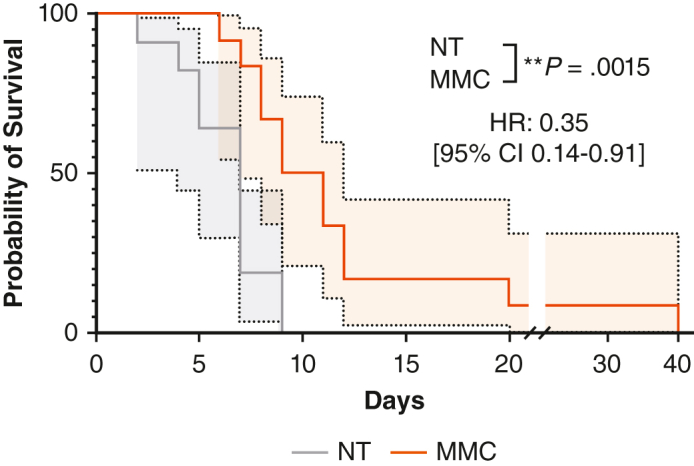
Sustainable MMC administration improves the survival of MPM-bearing mice.

**Central Message**

Diet supplementation with a novel microbiome modulator composition modifies the gut microbiota landscape and improves malignant pleural mesothelioma control through immune-modulating effects.

**Perspective**

Diet supplementation with a sustainably produced microbiome modulator composition could be a potent enhancer of anticancer immunity in the context of solid tumors in patients.

See Discussion on page 345.

Malignant pleural mesothelioma (MPM) is an aggressive cancer with a dismal prognosis strongly associated with exposure to asbestos or asbestos-like fibers. Its management remains challenging, with no effective treatment options available to date.

Apart from very specific and carefully selected clinical situations, the treatment of MPM is mostly based on systemic therapeutic approaches.^1^ The development of immunotherapy-based approaches in the past years has given hope and opened new prospects in the MPM field. Dual immune checkpoint inhibition in first-line therapy demonstrated significant improvements in overall survival compared with standard-of-care chemotherapy. Unfortunately, only a minority of patients responded to immunotherapy, with a 3-year overall survival of 23%, leaving significant room for further improvements.^2^ Potential explanations for poor responses of MPM to dual immune checkpoint inhibition could be their poor immunogenicity, their lack of activable antitumor T cells in the tumor bulk, the impaired function of effector T cells, and the lack of T memory cell formation.^3^ Therefore, overcoming these obstacles seems to be a way to make immunotherapies more effective.

Recently, several studies have highlighted the connection between the gut or tumor microbiota composition and immune checkpoint inhibitor (ICI) responsiveness of different cancer types such as colorectal, non–small cell lung cancers, and melanoma.^4^, ^5^, ^6^ The modulation of the gut microbiota was shown to favorably reshape the tumor immune landscape and improve immunotherapy efficacy. In-depth analyses of the 16S rRNA gene amplicon sequencing of the fecal microbiota of patients with cancer have shown that the enrichment in specific bacterial strains was associated with increased tumor infiltration by cytotoxic T cells and enhanced tumor responses to immune checkpoint blockade.^5^, ^6^, ^7^ An enhanced activity of the CD8+ T cells by short-chain fatty acids (SCFAs) that are metabolites derived from the gut microbiota is thought to play an important role in the improved tumor control.^8^ Furthermore, the presence of different strains was shown to correlate with the development of adverse reactions to immune- and chemotherapies, although the specific taxa are not, to date, elucidated.^9^

Therefore, it seems that the modulation of the microbiota through probiotics (living micro-organisms that, when administered in adequate amounts, confer a health benefit on the host) or prebiotics (fermentable nondigestible oligosaccharides or other components selectively used by beneficial gut bacteria, conferring a health benefit) may be a rational approach to improve immunotherapy efficacy. However, the use of prebiotics has been hampered by the important amounts of these substances required to induce significant changes in the gut microbiota composition.^10^^,^^11^

In the present study, we tested the impact of EMB008, a novel prebiotic microbiome modulator composition (MMC) derived from *Saccharomyces cerevisiae* yeast on the immune-mediated tumor control in an orthotopic murine model of MPM. Preliminary experiments in poultry suggest MMC to be effective in the milligram range per kilogram a day (unpublished data). We thus hypothesized that low dosing of MMC could have an impact on tumor progression through alterations in gut microbiome and associated modulation of immune response (see Figure 1 for a graphical abstract of the study).

**Figure 1:**
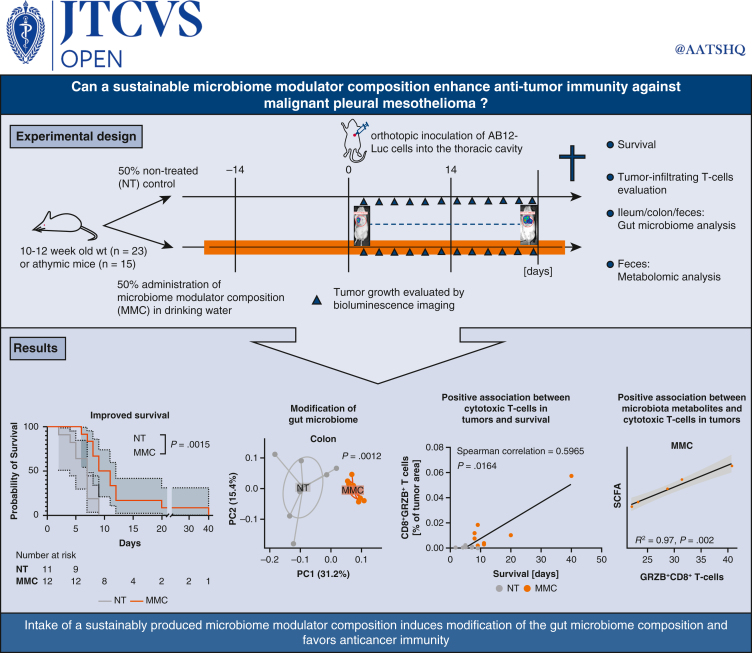
The impact of a microbiome modulator composition (MMC), a new generation prebiotic, on the gut microbiome and anticancer immunity was assessed in a syngeneic mouse model of malignant pleural mesothelioma. MMC was administered 2 weeks before cancer cell inoculation. Tumor growth was monitored by bioluminescence imaging. At the time of animal euthanasia, tumor, gut, and feces samples were collected for immune, microbiome and metabolomic analyses.

## Methods

Appendix E1 is available online with this article.

### Microbiome Modulator Composition

EMB008 is a novel MMC provided by Embion Technologies SA and obtained with Embion's proprietary extraction platform by processing food industry's byproduct. The MMC composed of oligomeric carbohydrate macromolecules and proteins. Importantly, EMB008 does not include any living organism (probiotic). The exact composition of the MMC is the property of Embion. The MMC was provided by Embion in the form of a dehydrated powder and was prepared at 31 ppm (7.75 mg of EMB008 in 250 mL of drinking water) in the drinking water of mice, corresponding to an estimated daily intake of 6.2 mg/kg body weight per day.

### Animal and Tumor Model

#### Housing and treatment assignation

**Figure E1:**
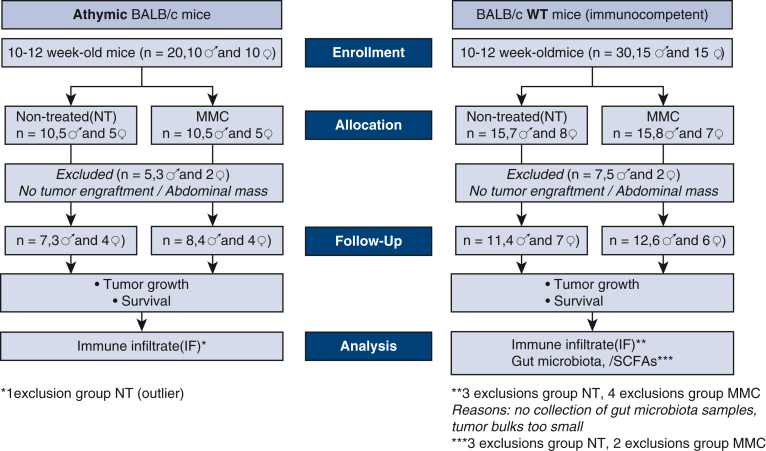
Flow chart of the experimental design research. *WT*, Wild-type; *NT*, not treated; *MMC*, microbiome modulator composition.

Animal experiments were initiated on 10- to 12-week-old BALB/c or BALB/c athymic mice imported from Charles River Laboratories (1:1 male-to-female ratio). All animals were kept in a specific pathogen-free environment, which included filtered air, sterilized food, water, bedding, and cages. The animals were acclimated for at least 1 week before the beginning of experiments, and all experiments were conducted in accordance with the Animal Welfare Act and the National Institutes of Health “Guidelines for the Care and Use of Laboratory Animals” and approved by the Committee for Animal Experiment for the Canton Vaud, Switzerland (authorization VD3345). After the acclimation period, animals were randomly assigned to no treatment or MMC groups with a sex ratio of males to females of approximatively 50:50. MMC was administered through drinking water 2 weeks before inoculation with cancer cells and continued to be administered throughout the course of the study. Drinking water was replaced every week. The experimental design is shown as a flow diagram in Figure E1.

#### Orthotopic tumor model

Mice were anaesthetized with a mix of ketamine/xylazine (80/10 mg/kg) and placed in a supine position. Then, 2.5 × 10^5^ AB12-luc cells resuspended in 50 μL of Dulbecco's Modified Eagle Medium without serum were injected through the fourth intercostal space using a 29-gauge needle inserted about 5 mm into the left pleural cavity. Tumor growth was recorded using bioluminescence imaging. Tumor growth curves and Kaplan–Meier curves were started when the tumors reached a volume associated to a photon flux ≥10^7^, which corresponds to the beginning of the tumor exponential growth phase. After tumor cell inoculation, animals were monitored daily and humanely killed if they presented a weight loss of more than 15% compared with the start of the experiment, rapid respiration, or significant decreased activity (human end points).

### 16S rRNA Gene Library Preparation and Amplicon Sequencing

Amplicon sequencing targeted the V1-V2 region of the 16S rRNA gene with primers F-27 and R-338 (see Table E1 for full sequences and Appendix E1 for details). Amplification was performed using the AccuPrime Taq DNA Polymerase High Fidelity kit (Invitrogen). No-template polymerase chain reaction controls (N = 2) were included. Libraries were loaded onto an Illumina MiSeq using pairwise chemistry, generating 250 × 2 read lengths (Lausanne Genomic Technologies Facility, University of Lausanne).

### Statistical Analysis

Kaplan–Meier curves and immunostainings statistical analyses were performed using GraphPad Prism, version 9.1.0, for Windows (GraphPad Software). Kaplan–Meier curves were compared using log-rank test and a difference in survival distribution was assumed when *P* ≤ .05. Hazard ratio and 95% confidence interval were calculated using the log-rank method. For immunostainings and SCFA comparisons, a 2-tailed Student *t* test was applied to assess differences in the distribution of untreated versus MMC-treated samples when a normal distribution was observed. A Welch's correction was applied to correct for unequal variance when required (for CD3/CD8, CD8/programmed death-1 [PD-1], CD8/cytotoxic T-lymphocyte associated protein-4 [CTLA-4], SCFA propionic acid). For normally distributed values, data were expressed as mean ± standard deviation. When the dataset did not satisfy normality (CD3/CD4 and M2 macrophages) Mann–Whitney *U* test was used and median and interquartile range were reported.

For gut microbiome analysis, outliers were mathematically identified by performing linear regressions and measuring the distance of each point from its corresponding fitted point (hat values). Samples with hat values 2 times greater than the average influence were considered influential and subsequently removed. Statistical analysis on community compositions were performed by calculating group centroids and performing a permutational multivariate analysis of variance. Subsequently, group and homogeneity of multivariable dispersions were performed, and a Tukey's honestly significant difference test was performed. For relative abundance comparisons, a Wilcoxon signed-rank test was performed.

### Data Availability

The data generated in this study are available upon request from the corresponding author.

## Results

### Tumor Immune Microenvironment in MMC-Treated Mice

**Figure 2:**
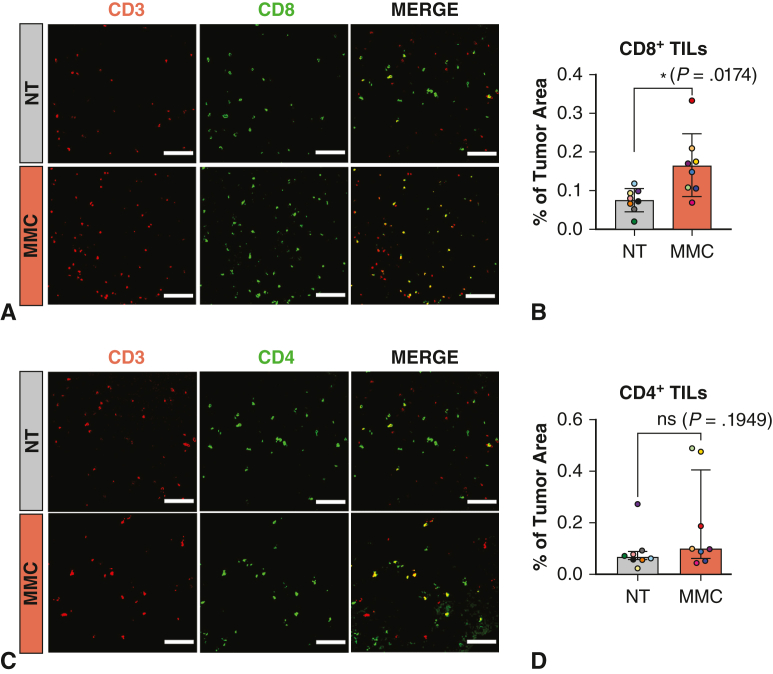
Impact of microbiome modulator composition (*MMC*) on T-cell infiltration into tumors. A and C, Representative images of CD8^+^ or CD4^+^ cells (*green*) costained with the lymphocyte marker CD3 (*red*) in tumors for nontreated (*NT*) and MMC-treated groups. Colocalization appears in *yellow*. Scalebar: 100 μm. B and D, Quantification of the colocalization area between CD3 and CD8 signal (B), CD3 and CD4 signal (D) normalized to tumor area. Mean ± standard deviation (*SD*) are represented on the graph for CD3 and CD8 colocalization (B) with the mean represented by the *horizontal line*. The *upper error bar* represents the mean value plus SD and the *lower error bar* the mean value minus SD. *P* values were calculated by using unpaired *t* tests with Welch correction for unequal variance. For CD3 and CD4 colocalization (D), the median and interquartile range are represented on the graph, with the median represented by the *horizontal line*, the 25th percentile by the *border of the lower error bar*, and the 75th percentile by the *border of the upper error bar*. *P* values were calculated using Mann–Whitney *U* test, as the values were not normally distributed. ∗*P* ≤ .05.

**Figure 3:**
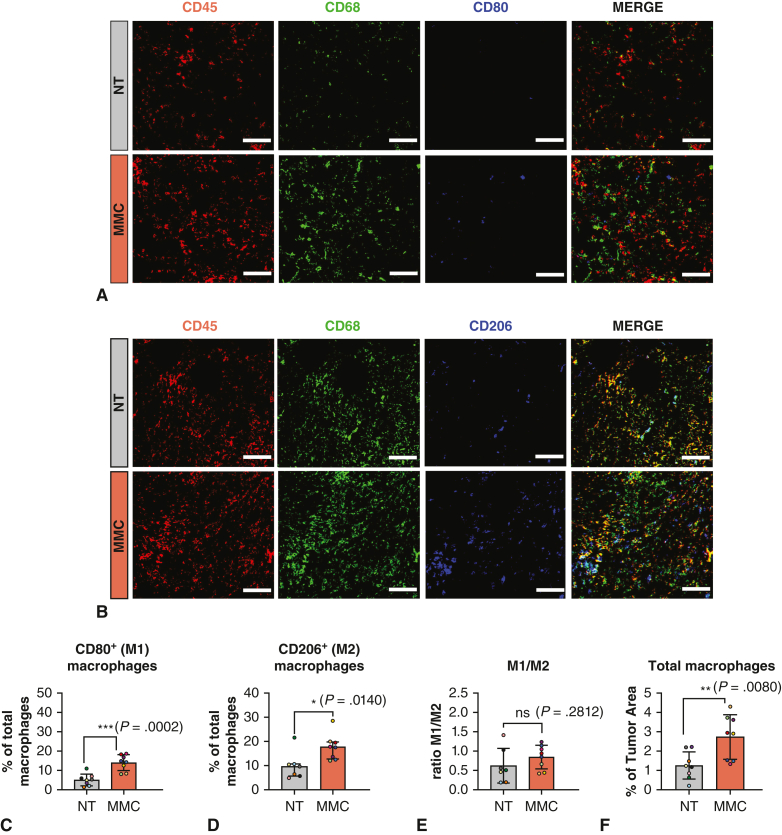
Impact of microbiome modulator composition (*MMC*) on tumor macrophages. A and B, Representative images of CD45^+^ (*red*) CD68^+^ cells (*green*) costained with the activation marker (M1-like macrophages) CD80 or the M2-like marker CD206 (*blue*) for nontreated (*NT*) and MMC-treated group. Scalebar: 100 μm. C and D, Quantification of the number of M1-like macrophages (C) and M2-like macrophages (D) normalized to tumor area. E, Ratio M1-like/M2-like macrophages. F, Quantification of the total amount of infiltrating macrophages (CD45^+^CD68^+^) normalized to tumor area. For M2-like macrophages quantification, the graph represents the median and interquartile range, with the median represented by the *horizontal line*, the 25th percentile by the *border of the lower error bar*, and the 75th percentile by the *border of the upper error bar*. *P* value was calculated using Mann–Whitney *U* test, as the values were not normally distributed. For the other quantifications, graphs represent mean ± standard deviation (*SD*) are represented on the graphs with the mean represented by the *horizontal line*. The *upper error bar* represents the mean value plus SD and the *lower error bar* the mean value minus SD. *P* values were calculated by using unpaired *t* tests. ∗*P* ≤ .05, ∗∗*P ≤* .01, ∗∗∗*P* ≤ .001.

Given the reported modulatory role of prebiotics in antitumor immunity, the impact of MMC intake on the immune microenvironment composition was first evaluated in our syngeneic orthotopic MPM mouse model. Particular attention was paid to T lymphocytes due to their crucial role in the antitumor response and macrophages because of their predominance in MPM tumors.^12^ MMC treatment revealed a significant increase in the content of effector CD3^+^CD8^+^ T lymphocytes in the bulk of MPM tumors whereas CD3^+^CD4^+^ T helper lymphocytes were unchanged (Figure 2, *A-D*). In addition, MMC significantly increased the overall amount of CD45^+^CD68^+^ macrophages. However, no difference in fold-increase was noticed between CD80^+^ M1-like and CD206^+^ M2-like macrophages, leaving the M1/M2 ratio unchanged (Figure 3, *A-F*).

**Figure 4:**
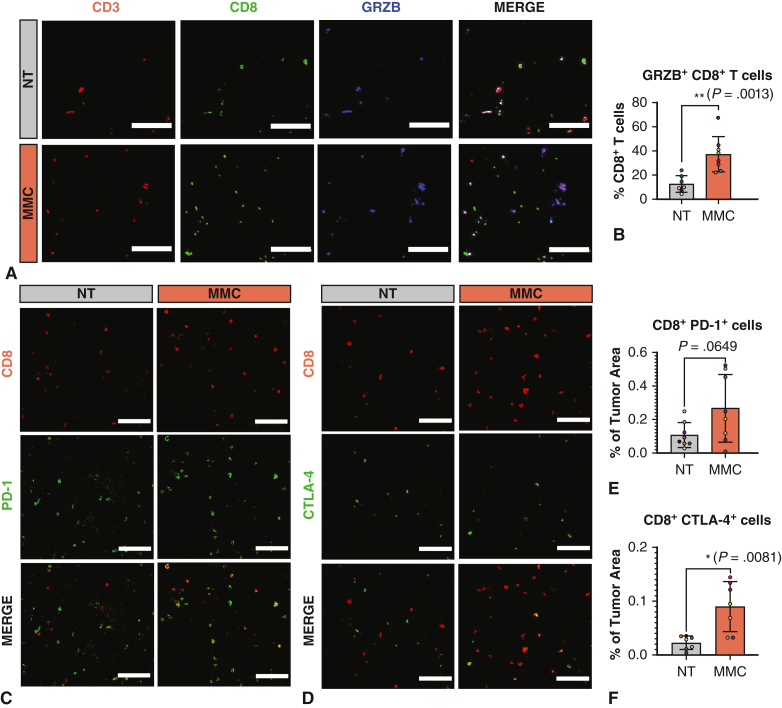
Impact of microbiome modulator composition (*MMC*) on T-cell activation and exhaustion. A, Representative images of GRZB (*blue*) staining and colocalization with CD3 (*red*) and CD8 (*green*) for nontreated (*NT*) and MMC-treated group. Scalebar: 100 μm. Colocalization signal appears in *white*. Scalebar: 100 μm. B, Quantification of the percentage of granzyme B^+^ CD8^+^ CD3^+^ lymphocytes. Mean ± standard deviation (*SD*) are represented on the graphs, with the mean represented by the *horizontal line*. The *upper error bar* represents the mean value plus SD and the *lower error bar* the mean value minus SD. *P* values were calculated by using unpaired *t* tests. ∗∗*P* < .01. C-F, The impact of prebiotic composition on PD-1 and CTLA-4 immune checkpoint molecules expression associated with CD8^+^ cells. C and D, Representative images of nontreated (*NT*) and the MMC-treated tumors stained for CD8 (*red*) and PD-1 or CTLA-4 (*green*). Colocalization appears in *yellow*. Scalebar: 100 μm. E and F, Quantification of immunofluorescence stainings. E, Colocalization area between CD8 and PD-1 signal and F, CD8 and CTLA-4 signal normalized by tumor area. Mean ± SD are represented on the graphs with the mean represented by the *horizontal line*. The *upper error bar* represents the mean value plus SD and the *lower error bar* the mean value minus SD. *P* values were calculated by using unpaired *t* tests with Welch correction for unequal variances. ∗*P* ≤ .05.

To understand whether CD8 T cells infiltrating the tumor upon MMC treatment have the capacity to recognize and kill cancer cells, the activation and exhaustion status of those cells was assessed by studying the expression of the cytotoxic cell granule protein granzyme B and the exhaustion markers PD-1 and CTLA-4. Of interest, an increased proportion of CD8 T cells expressing granzyme B in the MMC-treated tumors indicating cytotoxic activity (Figure 4, *A* and *B*). In addition, the presence of the exhaustion markers PD-1 and CTLA-4 associated with CD8 cells was also substantially enhanced in the MMC group compared with controls (Figure 4, *C-F*).

### MMC Delayed Tumor Progression and Extended Survival in Immunocompetent but Not in Athymic Mice

**Figure 5:**
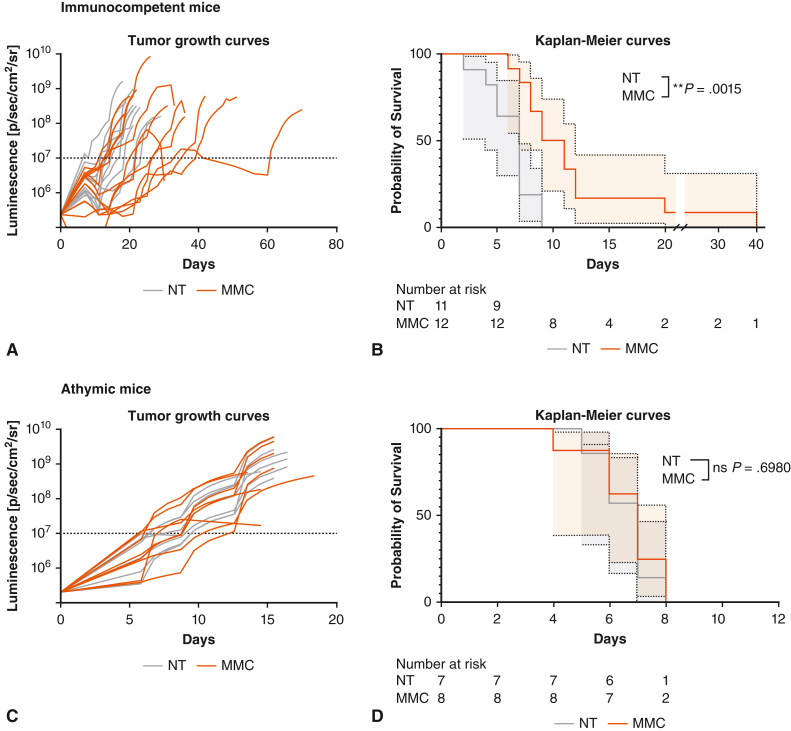
Impact of microbiome modulator composition (*MMC*) on tumor growth and animal survival in immunocompetent versus athymic mice. Tumor growth curves and survival for nontreated (*NT*, *gray*) and MMC-treated (*orange*) immunocompetent (A-B) and athymic (C-D) animals. A, Tumor growth curves measured by luminescence in photon per second per square centimeter per steradian (p/s/cm2/sr). Day 0 corresponds to a bioluminescence value of 1 to 4 × 10^7^ p/s/cm2/sr. B, Kaplan–Meier curves. Day 0 correspond to a bioluminescence value of 1 to 4 × 10^7^ p/s/cm2/sr. Hazard ratio (log-rank: 0.3532; 95% CI, 0.1365-0.9140) in immunocompetent animals and 0.8691 (95% CI, 0.3129-2.4140) for athymic animals. *P* values were calculated by using the log-rank test. ∗∗*P* ≤ .01. *CI*, Confidence interval.

**Figure E2:**
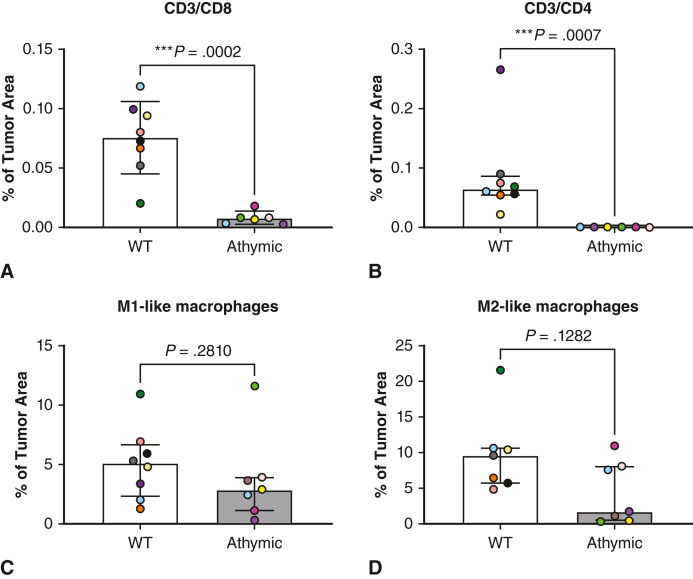
Quantification of (A) CD3/CD8, (B) CD3/CD4 T-cell infiltrates, and (C) M1-like and (D) M2-like macrophages in tumor sections of WT and athymic mice. Mean ± standard deviations are indicated on the graph of CD3/CD8, and *P* value was calculated by using unpaired *t* test. CD3/CD4, M1-like, and M2-like data sets were not normally distributed, and the graph show the medians and interquartile range. *P* values were calculated by using Mann–Whitney *U* test. *WT*, Wild-type.

**Figure E3:**
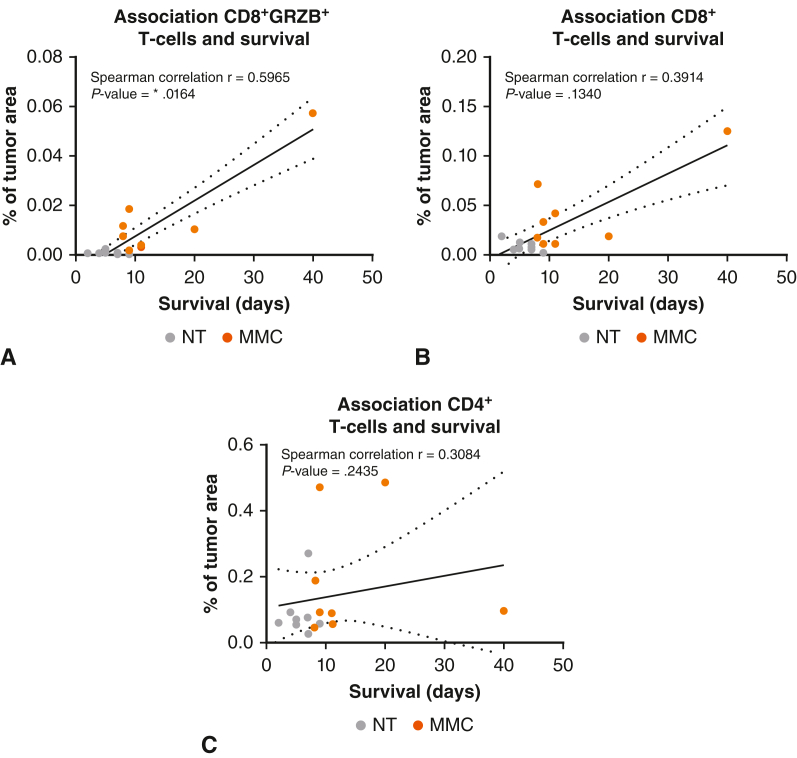
Association between tumor infiltrating CD8^+^GRZB ^+^ T cells, CD4 T cells, and animal survival. A positive association was found between (A) level of CD8^+^GRZB^+^ T cells and animal survival, whereas no significant association was observed between the amount of (B) CD8 T cells or (C) CD4 T cells and survival. ∗*P* ≤ .05.

To evaluate the impact of MMC on tumor control, we compared tumor growths in MMC-treated versus untreated immunocompetent animals. As shown in Figure 5, *A*, tumor growth was decreased in the MMC group compared with controls. The delay in tumor growth was correlated with a significant enhancement in survival time with median survival of 10 versus 7 days for MMC compared with untreated group (Figure 5, *B*) with a hazard ratio of 0.35 (95% confidence interval, 0.14-0.91). Of note, no impact on tumor engraftment was recorded. To assess the contribution of T cells in the observed response, we repeated the experiment in athymic mice of the same background that lack T cells. In such immunodeficient mice, MMC had no impact on tumor growth and mouse survival (Figure 5, *C* and *D*). Evaluation of the tumor immune microenvironment in athymic animals by immunofluorescence staining confirmed almost complete depletion of T cells, whereas the macrophage composition remains unaltered compared to wild-type animals (Figure E2). Finally, the relationship between MMC-related immunomodulatory effects and survival was assessed by calculating the Spearman correlation coefficient. A positive and significant correlation between the amount of CD8^+^ granzyme B (GRZB)^+^ T cells in tumors and the survival of the animals with a Spearman coefficient of 0.5965 (*P* value: .0164) was found, whereas we observed no significant association between CD8^+^ or CD4^+^ T cells infiltration and survival (Figure E3, *A-C*).

### MMC Promotes a Specific Gut Microbiome Composition Dependent on the Sampling Site

**Figure 6:**
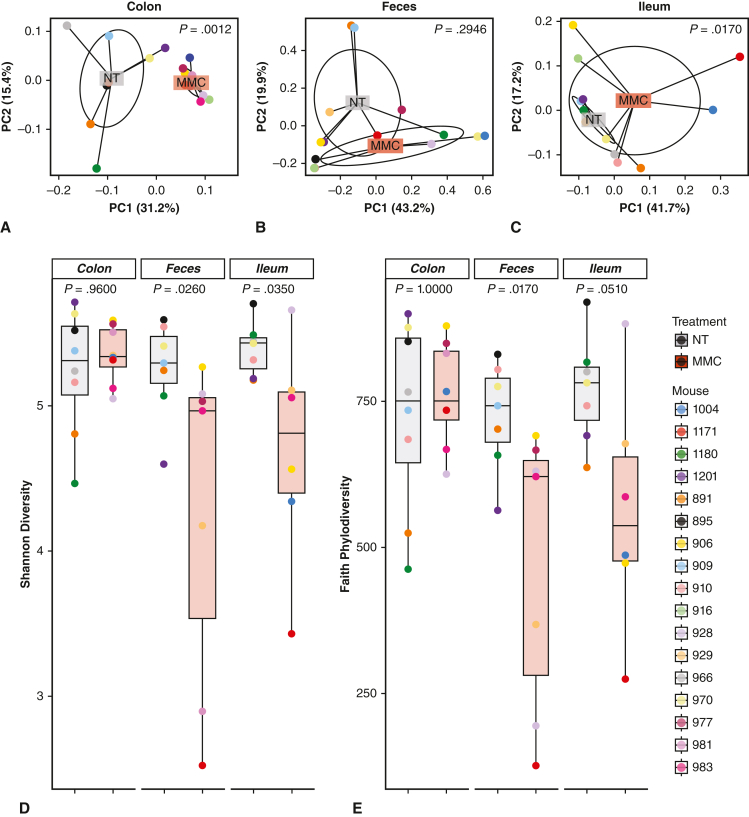
Impact of microbiome modulator composition (*MMC*) on gut microbiome community composition and diversity. A-C, Principal component analysis depicting the differences in community composition between nontreated controls (*NT*; *gray*) and MMC-treated (*orange*) subjects. Distance matrixes were calculated using weighted UniFrac. The community composition of the MMC-treated subject is altered compared with nontreated mice. MMC-treated subjects have a significantly different microbial community composition in the (A) colon (*P* = .0012), (B) feces (*P* = .2946), and (C) ileum (*P* = .017) compared with the nontreated controls. MMC-treated subjects have a decrease in beta-diversity (relative abundance between communities) in the colon, as evidenced by the decrease in dispersion. Permutational multivariate analysis of variance (ie, ADONIS) was performed to followed by the Tukey's honestly significant difference test. *P* values indicated the significance adjusted for variance variations between groups. D and E, Alpha diversity analyses of untreated controls (NT; *gray*) and MMC-treated (*orange*) subjects across all sampling locations (ie, ileum, colon and feces). D, Shannon diversity is significantly changed in the feces and ileum. E, Faith's phylodiversity is significantly reduces in fecal samples of MMC-treated subjects. The *lower and upper borders of the box-and-whisker plots* represent the 25th and 75th percentile respectively. The median is represented by the *middle horizontal line*. The *lower and upper whiskers* represent the minimum and maximum values of nonoutliers. *Extra dots* represent outliers. Indicated *P* values were obtained by applying a Wilcoxon signed-rank test.

The impact of MMC supplementation on microbiome composition in the ileum, colon, and feces was assessed following 16S rRNA amplicon sequencing. Principal coordinate analysis of community composition in the 3 different sampling sites revealed that the microbial composition in the colon and ileum of MMC-treated mice was statistically different (*P* value: .0012 and .0170, respectively) when compared with nontreated animals (Figure 6, *A-C*). The most pronounced changes in community composition occurred in the colon (Figure 6, *A*). Further analysis indicates a loss of the gut microbiome diversity with a significant decrease of the Shannon diversity index (taking in account species richness and relative abundance or evenness) observed in the fecal and ileum samples of the MMC-treated group (Figure 6, *D*; *P* values: .0260 and .0350, respectively). Faith's phylodiversity assessment provided similar results; however, although the *P* value obtained for ileum samples was very close to the significance threshold of .05, statistical significance was only found in fecal samples (Figure 6, *E*; *P* value: .0510 and .0170, respectively).

**Figure 7:**
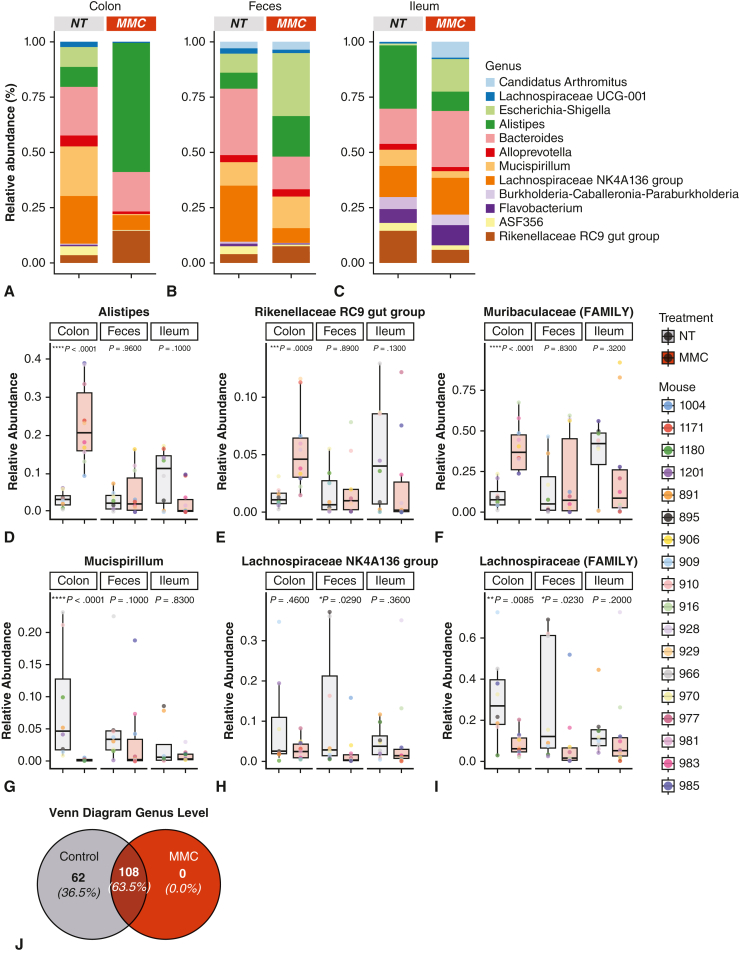
Impact of the prebiotic composition microbiome modulator composition (*MMC*) on the relative abundance of specific gut microbiota taxa. A-C, Relative abundance plot by sampling site (A) colon, (B) feces, and (C) ileum. Top 12 most abundant genera shown in the nontreated (*NT*) and MMC composition groups. MMC supplementation has a clear effect on relative ratios of the selected genera. D-I, Relative abundance per sampling site (ie, colon, feces, and ileum) for nontreated (“*NT*”: *gray*) and MMC-treated (*orange*), showing the selective enrichment of specific taxa by MMC supplementation. The *lower and upper borders of the box-and-whisker plots* represent the 25th and 75th percentile respectively. The median is represented by the *middle horizontal line*. The *lower and upper whiskers* represent the minimum and maximum values of nonoutliers. *Extra dots* represent outliers. Analyses were performed on D, Alistipes; E, the *Rikenellaceae* RC9 gut group; F, *Muribaculaceae* (FAMILY); G, *Mucispirillum*; H, the Lachnospiraceae NK4A136 group; and I, Lachnospiraceae (FAMILY). MMC-treated colon samples show the largest change compared with the nontreated controls. *P* values were calculated by using a Wilcoxon signed-rank test: ∗*P* ≤ .05, ∗∗*P* ≤ .01, ∗∗∗*P* ≤ .001, ∗∗∗∗*P* ≤ .0001. J, Venn diagram indicating the number of genera found in at least one subject in nontreated (“Control”: *gray*) and MMC-treated (*orange*) groups across all sample types. Of the total 170 genera identified, 108 were present in all groups. No unique taxa were found in the prebiotic-treated group, indicating that MMC may exert a selection pressure on the microbial population.

Comparison of community composition based on relative abundance of the top 12 genera showed a differential response based on sample type (Figure 7, *A-C*). More specifically, MMC treatment led to an enrichment of *Alistipes* and *Rikenellacaea RC9* gut group in the colon (Figure 7, *D* and *E*). In addition, we observed a significant enrichment in *Muribaculaceae* at the family level following MMC treatment (Figure 7, *F*). In contrast, other taxa were diminished by MMC administration. A significant reduction was observed in *Mucispirillum* genus (colon), Lachnospiraceae NK4A136 group (feces), and Lachnospiraceae family (colon and feces) (Figure 7, *G-I*). Interestingly, when assessing the total number of genera observed in the different groups, we observed that MMC treatment promotes the selection of specific taxa (Figure 7, *J*). When all sampling locations are considered (ie, colon, ileum, and feces), all genera observed in the MMC samples also appeared in the control samples. However, we observed a loss of 62 genera in the MMC samples (Table E2).

### The Presence of T-Cell Activation Marker Granzyme B Correlates With an Increase in Short-Chain Fatty Acid Levels in MMC-Treated Mice

**Figure 8:**
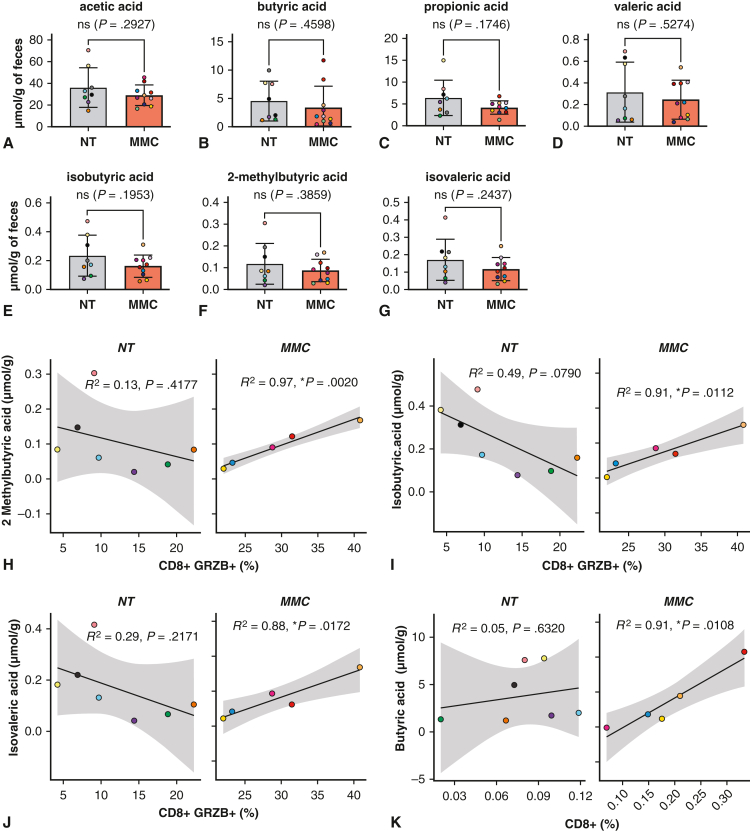
Impact of microbiome modulator composition (*MMC*) on fecal short-chain fatty acid (*SCFA*) level and association with the proportion of CD8+GRZB + T cells found in tumors. A-G, Level of SCFA in fecal samples of untreated controls (NT) and MMC-treated mice. Graphs show the mean ± standard deviation (*SD*) with the mean represented by the *horizontal line*. The *upper error bar* represents the mean value plus SD and the *lower error bar* the mean value minus SD. *P* values were calculated by using unpaired *t* tests or unpaired *t* tests with Welch correction (for propionic acid) to correct for unequal variances. H-K, Linear correlation of SCFAs concentrations (μmoL/g) with CD8^+^GRZB^+^ T cells into tumors (% of granzyme B^+^ CD8+ T-cells). Nontreated controls are depicted in *gray*, and the MMC-treated group is depicted in *orange*. The supplementation of MMC alters the relationship between SCFAs levels in feces and presence of granzyme B^+^ CD8^+^ T cells in tumors^.^ In the MMC-treated group, greater levels of granzyme B^+^ CD8^+^ T-cells coincide with greater levels of fecal SCFAs. H, 2-Methylbuteric acid; I, isobutyric acid; and J, isovaleric acid show strong correlations with R^2^ values of 0.97, 0.91 and 0.88 respectively. Butyric acid (K) shows strong correlations with R^2^ values of 0.91 with the level of CD8^+^ in tumors. All *P* values from linear regression analysis are indicated on the top of the graph (∗*P* ≤ .05).

Metabolite analysis in fecal samples of control and MMC-treated mice showed no differences in SCFA levels between the treatment groups (Figure 8, *A-G*). However, linear regression analyses show a strong correlation between fecal SCFA levels and CD8^+^GRZB^+^ T-cell infiltration in tumors (Figure 8, *H-K*). MMC-treated mice show a positive and statistically significant correlation between presence of CD8^+^GRZB^+^ T cells and of 2-methylbuteric acid isobutyric acid and isovaleric acid (Figure 8, *H-J*). Most strikingly, the relationship between the presence of CD8^+^GRZB^+^ and isobutyric acid, 2-methylbuteric acid, and isovaleric was inverted in MMC-treated subjects compared with controls (Figure 8, *H-J*). Furthermore, a positive correlation between CD8^+^ T-cell infiltration and butyric acid was found (Figure 8, *K*).

## Discussion

In the present study, we show the beneficial impact of a novel prebiotic MMC on the immune-mediated tumor control of a syngeneic MPM mouse model. Based on the low concentration of the MMC and the daily consumption of water by mice, very small quantities of MMC were required to improve MPM control and mouse survival. Importantly, no adverse effects of MMC administration were observed in mice, including body weight and stool appearance.

In recent years, the connection between microbiota and immunity has become clear.^13^ These findings have opened new perspectives for cancer management, suggesting that modulation of the microbiota could serve as a minimally toxic approach to enhance antitumor immunity and improve the response to ICI therapy. Successful approaches to modulate the gut microbiome have included fecal microbial transplantation^14^^,^^15^ or diet supplementation of probiotics/prebiotics. The probiotic supplementation showed mixed results, possibly due to microbiota dysbiosis and a decreased infiltration of tumors by cytotoxic CD8^+^ and helper CD4^+^ T cells.^7^ Mucin and inulin prebiotic supplementation, in contrast, showed improved antitumor immunity through dendritic and T-cell activation in melanoma and colon cancer mouse models.^16^ However, the translation of this concept in humans would require the administration of important quantities of prebiotics per day in the range of several dozens of grams. Our results showed that MMC does not appear to require high levels to be effective, making the concept plausible in patients. Although the exact reason for this result seems to depend on the composition of MMC, the exact mechanism has yet to be demonstrated. Further investigation of the timing of prebiotic exposure and its impact on tumor engraftment are still required to get closer to the patient setting.

Similarly with findings from other studies involving pro- and prebiotics, MMC demonstrated an enhancement in the infiltration of MPM with active CD8^+^ T cells.^17^^,^^18^ A correlation between enhanced CD8^+^GRZB ^+^ cytotoxic lymphocytes in the cancer microenvironment and tumor control was observed with MMC. Furthermore, the beneficial effects of MMC treatment on tumor growth and survival were abolished in T-cell–deficient mice.

Finally, the assessment of CD8 cells exhaustion status in MMC-treated animals, revealed an increase in these lymphocytes expressing PD-1 or CTLA-4 inhibitory checkpoint molecules compared to control. This result indicates that the pool of activatable and tumor-reactive CD8 lymphocytes is increased upon administration of MMC, which may suggest a potential benefit for tumor control of the MCM-ICI combination.

In addition to T-cell modulation, macrophages infiltration in the tumor of MMC group was significantly increased, but no apparent alterations in the ratio between M1-like and M2-like macrophages were detected. Moreover, no alteration in macrophages composition of tumors was observed in athymic animals compared to immunocompetent animals, indicating that macrophages do not contribute to the beneficial impact of MMC on tumor control.

Prebiotics have been reported to affect the function of immune cells either directly or indirectly in a microbiota dependent manner.^16^ Direct effects of prebiotics on immune cells have been reported to be associated predominantly with an immunosuppressive microenvironment, whereas improved effector T-cell function is more related to resident intestinal bacteria through production of SCFAs, a byproduct of prebiotic fermentation.^8^^,^^19^^,^^20^ MMC administration shows here a significant modification of the microbial community at 3 sample sites, including ileum, feces, and most prominently in the colon.

In particular, the SCFA-producing genera *Rikenellaceae* RC9 gut group and *Alistipes* were both enriched in the colon of MMC-treated mice. In addition, microbiome-derived SCFAs levels in fecal samples of MMC-treated mice were shown to be associated with increased level of cytotoxic T cells in tumors. Due to relatively low sample size and the limitations of 16S rRNA sequencing, we were unable to determine statistically significant associations between specific bacterial species and immune cell infiltration, GRZB colocation, and survival. Moreover, the exact mechanism by which MMC could selectively stimulate the growth and activity of favorable bacterial strains involved in the antitumor response remains to be determined. Functional studies may help to predict the response to the prebiotic treatment as it strongly relies on the composition of the intestinal microbiota.

Overall, our findings showed that a novel prebiotic MMC modulator was effective at low doses to modulate the immune microenvironment and improve MPM tumor control in a syngeneic tumor model.

Our study has certain limitations that should be highlighted. The impact of MMC on antitumor immunity was only assessed in a single MPM mouse model. The AB12 model is a biphasic MPM model and assessing the impact on pure epithelioid and sarcomatoid model of MPM would be useful in the future to understand which histotype might benefit from MMC supplementation. Furthermore, the infiltration of immune cells into tumors after MMC was only assessed by immunostaining of tumor sections. Validation of the immunostaining data and further characterization of the different immune subsets, including regulatory immune cells, by flow cytometry would provide a better understanding of the exact immune impact of MMC on tumor bulk. Finally, MMC was administered in the drinking water 14 days before inoculation of the cancer cells, and the effective dose of MMC was calculated on the basis of the average water consumption per animal per day. This experimental design is commonly used in preclinical studies in mice in order to limit the stress inflicted on the animal and circumvent the rapid progression of the tumor, which severely limits the observation window.^11^^,^^21^ Nevertheless, controlling the dose administered by oral gavage could help to measure more precisely the impact of the prebiotic on tumor progression and the tumor microenvironment. Prophylactic administration of prebiotic might also affect tumor microenvironment and response to treatment. Validation in a therapeutic setting would therefore be ideal from a translational point of view if the animal model allows.

Despite these limitations, our study is an initial proof-of-concept study suggesting that such strategy may be valuable in combination with immune checkpoint inhibition in patients bearing solid tumors. This supports the necessity for future trials with this compound in patients.

### Webcast

You can watch a Webcast of this AATS meeting presentation by going to: https://www.aats.org/resources/brewery-sludge-as-a-new-prebiotic-adjuvant-to-improve-anticancer-immunity-in-pleural-mesothelioma.

**Figure:**
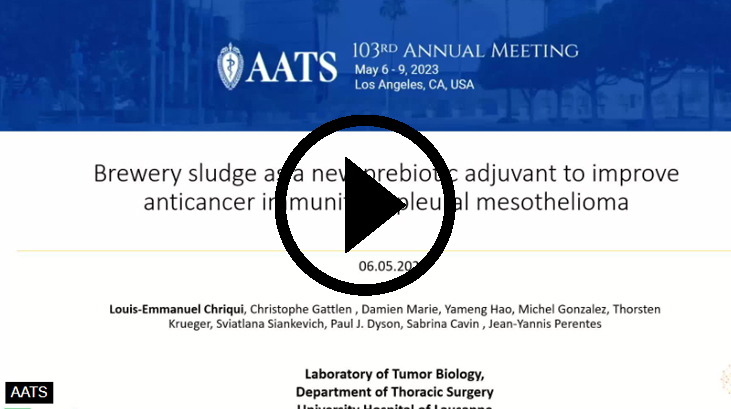

## Conflict of Interest Statement

S.S. is co-founder and employee of Embion Technologies SA, Switzerland, which developed and provided the MMC compound. However, the results were shared with Embion only at the end of the study so as not to interfere with the conduct of the study and interpretation of results. All other authors reported no conflicts of interest.

The *Journal* policy requires editors and reviewers to disclose conflicts of interest and to decline handling or reviewing manuscripts for which they may have a conflict of interest. The editors and reviewers of this article have no conflicts of interest.

## Figures and Tables

**Table E1:** Sequencing primers

Name	Method	Sequence (5′-3′)
27F	Illumina MiSeq	**AATGATACGGCGACCACCGAGATCTACAC***TATGGTAATTCCA*GMGTTYGATYMTGGCTCAG
338R	Illumina MiSeq	**CAAGCAGAAGACGGCATACGAGAT**NNNNNNNNNNNN*AGTCAGTCAGAA*GCTGCCTCCCGTAGGAGT
926F	qPCR	AAACTCAAAKGAATTGACGG
1062R	qPCR	CTCACRRCACGAGCTGAC

The V1-V2 hypervariable region of the 16S rRNA gene was amplified using the 27F and 338R Illumina sequencing primers. For load quantification, the V6 hypervariable region of the 16S rRNA gene was amplified with the aid of the qPCR primers 926F and 1062R. Illumina adapter sequences are indicated in bold, linkers are indicated in italic, and target sequences are underlined. Samples specific identification barcodes are denoted as NNNNNNNNNNN. *qPCR*, Quantitative polymerase chain reaction.

**Table E2:** List of lost genera

Kingdom	Phylum	Class	Order	Family	Genus
Bacteria	Acidobacteriota	Acidobacteriae	Bryobacterebs	Bryobacteraceae	*Bryobacter*
Bacteria	Actinobacteriota	Acidimicrobiia	Microtrichales	llumatobacteraceae	CL500-29 marine group
Bacteria	Actinobacteriota	Actinobacteria	Actinomycetales	Actinomycetaceae	*Actinomyces*
Bacteria	Actinobacteriota	Actinobacteria	Corynebacteriales	Corynebacteriaceae	*Lawsonella*
Bacteria	Actinobacteriota	Actinobacteria	Corynebacteriales	Mycobacteriaceae	*Mycobacterium*
Bacteria	Actinobacteriota	Actinobacteria	Corynebacteriales	Nocardiaceae	*Rhodococcus*
Bacteria	Actinobactenota	Actinobacteria	Frankiales	Geodenhatophilaceae	*Blastococcus*
Bacteria	Actinobactota	Actinobacteria	Frankiales	Sporichthyaceae	*Candidatus Planktophila*
Bacteria	Actinobacteriota	Actinobacteria	Micrococcales	Micrococcaceae	*Kocuria*
Bacteria	Actinobacteriota	Actinobacteria	Micrococcales	Sanguibacteraceae	*Sanguibacter*
Bacteria	Actinobacteriota	Actinobacteria	Propionibacteriales	Propionlbacteriaceae	*Cutibacterium*
Bacteria	Actinobacteriota	Actinobacteria	Propionibacteriales	Propionbactenaceae	*Naumannella*
Bacteria	Actinobacteriota	Actinobacteria	Streptomycetales	Streptomycetaceae	*Streptomyces*
Bacteria	Actinobacteriota	Coriobacteriia	Coriobacteriales	Atopobiaceae	*Atopobium*
Bacteria	Actinobacteriota	Coriobacterria	Coriobacteriales	Eggerthellaceae	*Parvibacter*
Bacteria	Bacteroidota	Bacteroidia	Chilinophagales	Chitinophagaceae	*Sediminibacterium*
Bacteria	Bacteroidota	Bacteroidia	Chltinophagales	Saprospiraceae	*Phaeodactylibacter*
Bacteria	Bacteroidota	Bacteroidia	Cylophagales	Spirosomaceae	*Dyadobacter*
Bactena	Bacteroidota	Bacteroidia	Flavobactenales	Croanitomicaceae	*Fluviicola*
Bacteria	Bacteroidota	Bacteroidia	Flavobacteriales	Weeksellaceae	*Chryseobacterium*
Bacteria	Bacteroidota	Bacteroidia	Sphingobacteriales	Sphingobacteriaceae	*Pedobacter*
Bacteria	Campilobacterota	Campytobactena	Campylobacterates	Arcobacteraceae	*Pseudarcobacter*
Bacteria	Deinococcota	Deinococci	Deinococcales	Trueperaceae	*Truepera*
Bacteria	Deinococcota	Deinococci	Thelmales	Thermaceae	*Thermus*
Bacteria	Firmicutes	Bacilli	Lactobacillales	Aerococcaceae	*Abiotrophia*
Bacteria	Firm icutes	Bacilli	Staphylococcales	Staphytococcaceae	*Jeotgalicoccus*
Bacteria	Firmicutes	Bacilli	Therrnicanales	Thermlcanaceae	*Thermicanus*
Bacteria	Firmicutes	Clostridia	Eubacteriales	Ana erofustaceae	*Ana erofustis*
Bacteria	Firm icotes	Clostridia	Lachnospira les	Lachnosprraceae	[*Eubacterium*] *ventriosum* group
Bacteria	Firmicutes	Clostridia	Monoglobales	Monolobaceae	*Monoglobus*
Bacteria	Firmicutes	Clostridia	Oscillospirales	Ruminococcaceae	*Subdoligranulum*
Bacteria	Firmicutes	Clostridia	Oscillospirales	Rumlnococcaceae	UBA1819
Bacteria	Firmicutes	Clostridia	Peplostreptococcales TissiereIlales	Anaerovoracaceae	[*Eubacterium*] *nodatum* group
Bacteria	Firmicutes	Clostridia	Peplostreptococcales Tissierellales	Anaerovoracaceae	Family XIII AD3011 group
Bacteria	Fusobacteriota	Fusobactedia	Fusobacteriaies	Leptotrichiaceae	*Leptotrichia*
Bacteria	Patescibacteria	Saccharimonadia	Saccha rimonadales	Sacchanmonadaceae	*Candidatus* Saccharimonas
Bacteria	Proteobacteria	Alphaproteobacteria	Caulobacterales	Caulobacteraceae	*Phenylobacterium*
Bacteria	Proteobacteria	Alphaproteobacteria	Paracaedibacterales	Paracaedibacteraceae	*Candidatus* Paracaedibacter
Bacteria	Proteobacteria	Alphaproteobacteria	Rhizobiales	Beijerinckiaceae	*Bosea*
Bacteria	Proteobacteria	Alphaproteobacteria	Rhizobiales	Beijerinckiaceae	*Chelatococcus*
Bacteria	Proteobactena	Alphaproteobacteria	Rhizobiales	Xanthobacteraceae	*Pseudolabrys*
Bacteria	Proteobactena	Alphaproteobacteria	Sphingomonadales	Sphingomonadaceae	*Porphyrobacter*
Bacteria	Proteobacteria	Alphaproteobacteria	Sphingomonadales	Sphingomonadaceae	*Oipengyuania*
Bacteria	Proteobacteria	Alphaproteobactena	Sphingomonadales	Sphingomonadaceae	*Sphingopyxis*
Bacteria	Proteobacteria	Gammaproteobacteria	Burkholderiales	Alcaligenaceae	*Parviblum*
Bacteria	Proteobacteria	Gammaproteobacteria	Burkhoideriales	Burkholderiaceao	*Cupriavidus*
Bacteria	Proteobacteria	Gammaproteobacteria	Burkhoideriales	Comamonadaceae	*Aquabacterium*
Bacteria	Proteobactena	Gammaproteobacteria	Burkholderiales	Comarnonadaceae	*Curvibacter*
Bacteria	Proteobacteria	Gammaproteobacteria	Burkholderiales	Comamonadaceae	*Hydrogenophaga*
Bacteria	Proteobacteria	Gammaproteobacteria	Burkholderiales	Comamonadaceae	*Inhella*
Bacteria	Proteobacteria	Gammaproteobacteria	Burkho4deriales	Comamonadaceae	*Polaromonas*
Bacteria	Proteobacteria	Gammaproteobacteria	Burkholderiales	Comamonadaceae	*Tepidimonas*
Bacteria	Proteobacteria	Gammaproteobacteria	Burkholderiales	Neissenaceae	*Neissena*
Bacteria	Proteobactena	Gammaproteobacteria	Burkhotderiales	Oxalobacteraceae	*Collimonas*
Bacteria	Proteobacteria	Gammaproteobacteria	Burkholderiales	Oxalobacteraceae	*Janthinobacterium*
Bacteria	Proteobacteria	Gammaproteobacteria	Burkholderiales	Oxalobacteraceae	*Massilia*
Bacteria	Proteobacteria	Gammaproteobacteria	Burkholderiales	Rhodocyclaceae	C39
Bacteria	Proteobacteria	Gammaproteobacteria	Burkholderiales	Rhodocyclaceae	*Ferribacterium*
Bacteria	Proteobacteria	Gammaproteobacteria	Burkholderiales	Rhodocyclaceae	*Zoogloea*
Bacteria	Proteobacteria	Gammaproteobacteria	Pseudomonadales	Moraxellaceae	*Enhydrobacter*
Bacteria	Proteobactena	Gamrnaproteobacteria	Xanthomonadales	Xanthomonadaceae	*Therrnomonas*
Bacteria	Spirochaetota	Leptospirae	Leptospirales	Leptospiraceae	*Tumeriella*

**Table E3:** Antibodies used for immunofluorescence staining of tumor section

Antibodies	Source	Reference	Dilution used
Primary			
Anti-CD3	GeneTex	GTX16669	1/100
Anti-CD4	BD Pharmingen	550,280	1/50
Anti-CD8a	BD Pharmingen	550,281	1/50
Anti-CD68	Cell Signaling Technology	97778S	1/200
Anti-Granzyme B	eBioscience	14-8822-82	1/100
Anti-CD45 - Cy5	BioLegend	103,109	1/100
Anti-CD80 - Brillant Violet 421	BioLegend	104,725	1/50
Anti-CD206 - Brillant Violet 421	BioLegend	141,717	1/100
Secondary			
Alexa Fluor 488 donkey anti-rabbit	Thermo Fisher Scientific	A-21206	1/300
Alexa Fluor 488 donkey anti-rat	Thermo Fisher Scientific	A-21208	1/300
Alexa Fluor 568 donkey anti-rabbit	Thermo Fisher Scientific	A-10042	1/300
Alexa Fluor 647 goat anti-rat	Thermo Fisher Scientific	A-21247	1/300

**Table E4:** List of targeted SCFA and corresponding SIM transitions

Metabolite	Retention time, min	SIM QUANT; m/z	SIM QUAL; m/z
Acetic acid	7.832	60	61
Formic acid	8.565	46	47, 48
Propionic acid	8.901	74	75, 76
Isobutyric acid	9.242	73	74, 88
Butyric acid	9.963	60	73, 74, 88
2-Methylbutyric + isovaleric acid	10.436	60, 74	74, 60
Valeric acid	11.218	60	73, 74
Crotonic acid	11.64	86	68, 69, 71
3-Methylvaleric acid	11.815	60	61, 73, 74
4-Methylvaleric acid	11.936	74	75, 75
Hexanoic acid	12.388	60	73, 74
2-Methylhexanoic acid	12.62	74	73, 75
4-Methylhexanoic acid	13.227	71	73, 74
2-Propylvaleric acid	13.343	102	103, 104
Heptanoic acid	13.498	60	101, 102

*SCFA*, Short-chain fatty acid.

**Table E5:** Contaminants identified using Decontam R package

Kngdom	Phylum	Class	Order	Family	Genus	Species
Bacteria	Firmicutes	Clostridia	Oscillospirales	Osallospraceae		
Bacteria	Firmicutes	Clostridia	Lachnospirales	Lachnospiraceae		
Bacteria	Actinobacteriota	Actinobacteria	Corynebacteriates	Nocardiaceae		
Bacteria	Actinobacteriota	Actinobacteria	Propionibacteriales	Propionibacteriaceae	*Cutibacterium*	
Bacteria	Bacteroidota	Bacteroidota	Flavobacteriales	VVeeksetlaceae	*Cloacibacterium*	
Bacteria	Proteobactena	Gammaproteobactena	Pseudomonadales	Pseudomonadaceae	*Pseudomonas*	monteilii
Bacteria	Firmicutes	Bacilli	RF39			
Bacteria	Firmicutes	Clostridia	Lachnospirales	Lachnospiraceae		
Bacteria	Actinobacteriota	Conobactenia	Coriobacteriales			
Bacteria	Bacteroidota	Bacteroidota	Flavobactenales	Flavobacteriaceae		
Bacteria	Actinobacteriota	Coriobactenia	Conobacteriales	Eggerthellaceae	*Enterorhabdus*	
Bacteria	Firmicutes	Clostridia	Lachnospirales	Lachnospiraceae	Lachnospiraceae UCG-001	
Bacteria	Firmicutes	Clostridia	Clostridia UCG014			
Bacteria	Firmicutes	Clostridia	Oscillospirales	Oscillospirales		
Bacteria	Firmicutes	Clostridia	Lachnospirales	Lachnospirales		
Bacteria	Firmicutes	Clostridia	Lachnospirales	Lachnospirales		

Contaminants were identified using the Decontam R package, version (1.20.0). First, the amount of 16s rRNA present in each sample was quantified by means of a qPCR using the V6 hypervariable region primers. Subsequently, the starting quantity of 16s rRNA was used to perform the combined, frequency and prevalence contaminant identification analysis. A total of 11 contaminants were Identified and removed before subsequent analysis was performed. *qPCR*, Quantitative polymerase chain reaction.

**Figure E4:**
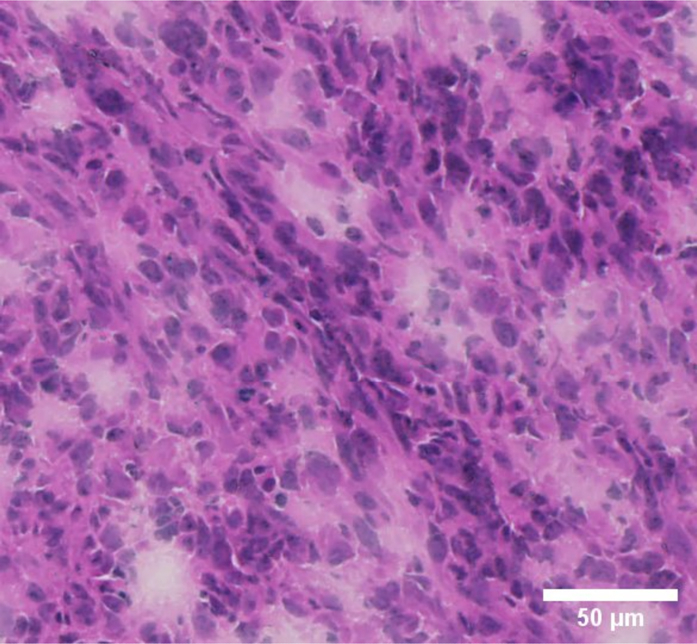
Hematoxylin and eosin staining of biphasic AB12 tumor. Most tumor area exhibits an epithelioid morphology with few spindle areas. Scale bar: 50 μm.

## Appendix E1. Cell Lines and Culture Conditions

Biphasic malignant mesothelioma cells AB12 (RRID:CVCL_4405) isolated from ascites of asbestos-exposed wild-type BALB/c mice^E1^ were transduced with a luciferase reporter gene to generate AB12-luc cells. Cells were grown in RPMI 1640 medium supplemented with 10% fetal bovine serum and 5 μg/mL puromycin and passaged at 80% of confluency. Most cells present a sarcomatoid morphology in culture,^E2^ but once inoculated in mice, these cells cause the development of a biphasic microbiome modulator composition with only few spindle cell areas (Figure E4).

## Bioluminescence Imaging

Tumor sizes were assessed by bioluminescence measurement using the In-Vivo Imaging System (IVIS) Lumina S5 every 3 days. One hundred microliters of VivoGlo Luciferin (15 mg/mL; Promega) were injected intraperitoneally 15 minutes before imaging and animals were kept under isoflurane anesthesia during the entire course of monitoring. The tumor sites showing bioluminescence signals were identified as the regions of interest and the total photon counts were quantified using the Living Image software.

## Immunofluorescence Staining of Tumor Section

At human end points, mice were euthanized by intraperitoneally injection of pentobarbital (150 mg/kg) and intracardiac perfusion with NaCl 0.9% was performed. Pleural tumors were collected, embedded in OCT, cut into 8-μm sections, and mounted on glass microscope slides. Tumor sections were then fixed at −20 °C in methanol for 10 minutes, washed 3 times in phosphate-buffered saline (PBS) and mounted on Shandon coverplate system.

Non-specific reactivity was blocked by incubating sections for 1 hour with blocking buffer (5% normal donkey serum, 0.1% bovine serum albumin, 0.3% Triton in PBS). Samples were then incubated overnight at 4 °C in blocking buffer containing primary antibodies, washed 3 times with PBS, and incubated for 1 hour at room temperature with secondary antibodies in blocking buffer. At the end of the procedure, sections were washed in PBS and mounted in Fluoromount-G mounting medium. Primary and secondary antibodies used are recapitulated in Table E3.

## Image Analysis

Tumor sections were scanned with a Zeiss Axioscan Z1 slide scanner at 10 × magnification. Image analysis was performed using ImageJ software. The following threshold values were used for the different staining: CD3: 65-150, CD4: 60-120, CD8: 50-120, CD45: 40-150, CD68: 20-180, CD206: 70-240, CD80: 10-70.

Colocalization data between CD4/CD3, CD8/CD3, and CD45/CD68 were obtained using the RG2B colocalization plugin. Sequential colocalizations were performed for granzyme B, CD206, and CD80. Colocalization data were normalized to tumor area, total amount of CD8^+^CD3^+^ T cells for granzyme B staining or CD45^+^CD68^+^ cells (macrophages) for CD206 and CD80 stainings.

## Gas Chromatography/Mass Spectrometry Analysis of Short Chain Fatty Acids

Short chain fatty acids from C2 to C7 including hydroxylated and methylated derivatives were separated in a DB-FFAP column (30 m × 0.25 mm i.d. × 0.25 μm; Agilent Technologies) and analyzed by single quadrupole mass spectrometer operating in SIM mode (EI 70 eV) using a 2-millisecond dwell time (Agilent Technologies; Table E4). Chromatographic conditions were set as follows: carrier gas He 1.2 mL/min at constant flow. Initial temperature was set at 40 °C, the temperature increment was 10 °C/min along the gradient until it reached 200 °C, and afterwards 40 °C/min until 240 °C (over 3 minutes).

Feces samples (10 mg) were homogenized with 1 mL of water. An aliquot (100 μL) of homogenate was extracted with 140 μL of methyl-tert-butyl ether spiked with the internal standard solution as previously described by Lotti and colleagues.^E3^ Samples were centrifuged for 15 minutes at 15,000 rpm (4 °C) and 1.5 μL of supernatant was injected in spitless mode (240 °C) into the gas chromatography/mass spectrometry system.

## Bacterial DNA Extraction

At the time of animal euthanasia, one fecal pellet, colon or ileum (without luminal content) from each mouse was collected into a sterile 1.5-mL Biopur tube (Eppendorf), placed on dry ice, and stored at −80 °C until further processing. Total bacterial DNA was isolated using the QiaAMP Fast DNA Stool Mini Kit (QIAGEN) according to the manufacturer's instructions. DNA was eluted with 200 μL of AE buffer (provided with the kit). DNA was stored at 4 °C until being used for library preparation.

## Bioinformatics Analysis of GUT Microbiome

All analyses were performed in R, version 4.2.1 (R Core Team [2021]. R: A language and environment for statistical computing; R Foundation for Statistical Computing; https://www.R-project.org). Demultiplexing, removal of chimeric and short reads, single base resolution of reads into amplicon sequence variants (ASVs) using the Divisive Amplicon Denoising Algorithm 2 algorithm^E4^ and taxonomic annotation using the SILVA database^E5^ were performed using a dedicated pipeline available at https://github.com/chuvpne/dada2-pipeline with default parameters. Taxonomic classification and exact sequence matching were performed using the SILVA database (version 123). Contaminants were identified using the Decontam R package version (1.20.0). First, the amount of 16S rRNA present in each sample was quantified by means of a quantitative polymerase chain reaction using the V6 hypervariable region primers. Subsequently, the starting quantity of 16S rRNA was used to perform the combined frequency and prevalence contaminant identification analysis. A total of 11 contaminants were identified and removed before subsequent analysis was performed (Table E5). ASV filtering and ordination analyses were performed using the phyloseq R package (version 1.42.0) and visualized using the ggplot2 R package (version 3.4.0). Data transformation and alpha diversity analyses were performed using the Microbiome (1.20.0) and Picante (1.8.2) R Packages. Single ASV reads and sample with a sequencing depth below 15,000 reads were removed before downstream processing was performed. The ASV count table then was normalized by both rarefication as well as Hellinger transformation, where the square root of each ASV count per sample is divided by the total read count for that specific ASV. Principle coordinate analysis was performed using weighted UniFrac distance matrix calculated using the vegan R package (version 2.6.4).
